# Data on RDM16 and STA1 regulate differential usage of exon/intron in RNA directed DNA Methylation pathway

**DOI:** 10.1016/j.dib.2017.03.050

**Published:** 2017-04-08

**Authors:** Ravi Datta Sharma, Bert Bogaerts, Neha Goyal

**Affiliations:** a Centre for Microbial and Plant Genetics, KU Leuven, 3000, Belgium; b Amity institute of Biotechnology and Amity institute of Integrative Sciences and Health, Amity University Haryana, NH-8, Panchgaon, Gurgaon, Haryana 122413, India; c School of Life Sciences, Jawaharlal Nehru University, New Delhi 110067, India

**Keywords:** RNA directed DNA methylation pathway, Alternative splicing, RNA-Seq, Arabidopsis thaliana

## Abstract

This article contains data on *RDM16* and *STA1* regulate differential usage of exon/intron in RNA directed DNA Methylation pathway (RdDM) (Sharma et al., 2016) [5]. This data include expression profiles of top 100 genes that has at least one exon or intron differentially expressed in three different contrast, i.e., WT (Wild type) *vs RDM16*, WT *vs STA1*, and *RDM16 vs STA1*. Also we included the alignment of *MORC6* protein to the ATPase-C family members that have conserved three ATP binding sites and conserved Mg2+ binding sites in the spliced exon.

**Specifications Table**

**Table:** 

Subject area	*Bioinformatics, Genomics*
More specific subject area	*Alternative splicing, Differential Expression*
Type of data	*Figures, Table, Alignment*
How data was acquired	Gene Expression Omnibus (GEO) id: GSE44635, URL: https://www.ncbi.nlm.nih.gov/geo/query/acc.cgi?acc=GSE44635 from the article [1]
Data format	*Analyzed* (Fig. 1, Fig. 2, Fig. 3*, Alignment*Fig. 4, Fig. 5, Table 1)
Experimental factors	*Secondary analysis of published data*
Experimental features	*Computational analysis*
Data source location	–
Data accessibility	*Accessible from this article*

**Value of the data**

• The data from article [5] shows the expression profiles of the genes that contain at least one alternative splicing event in different conditions. This information will be useful for other researchers to understand the regulation of gene expression by alternative splicing.
• Alignment of *MORC6* protein to the ATPase-C family simplifies the mechanism by which splicing factor RDM16 regulate the MORC6.
• This data provides the information of the genes that are affected in RdDM pathway by knockdown of RDM16 and STA1 splicing factors. This data will help other researcher to validate the findings of the exon/intron level analysis in RdDM pathway.

## 1 Data

**Fig. 1:**
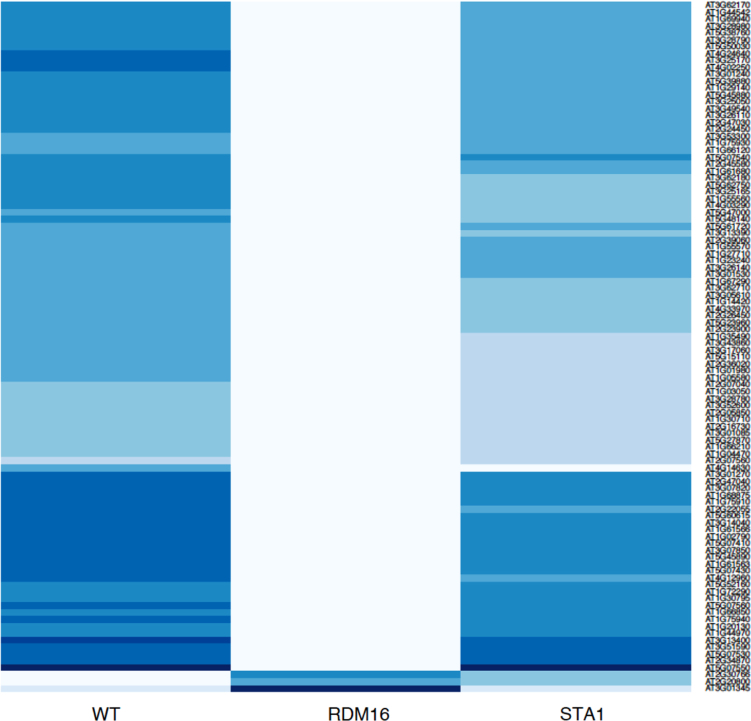
This figure depicts expression profile of top 100 genes that has at least one exon or intron differentially expressed in WT vs RDM16. Color key used in expression profiles of genes in different contrasts is given with Fig. 3. (For interpretation of the references to color in this figure legend, the reader is referred to the web version of this article.)

**Fig. 2:**
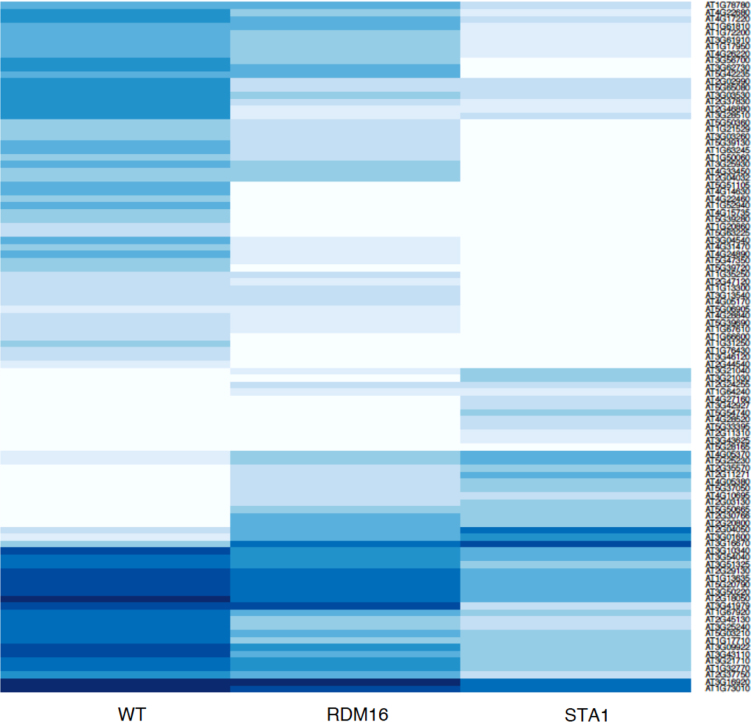
Depicts expression profile of top 100 genes that has at least one exon or intron differentially expressed in WT vs STA1. Color key used in expression profiles of genes in different contrasts is given with Fig. 3.

**Fig. 3:**
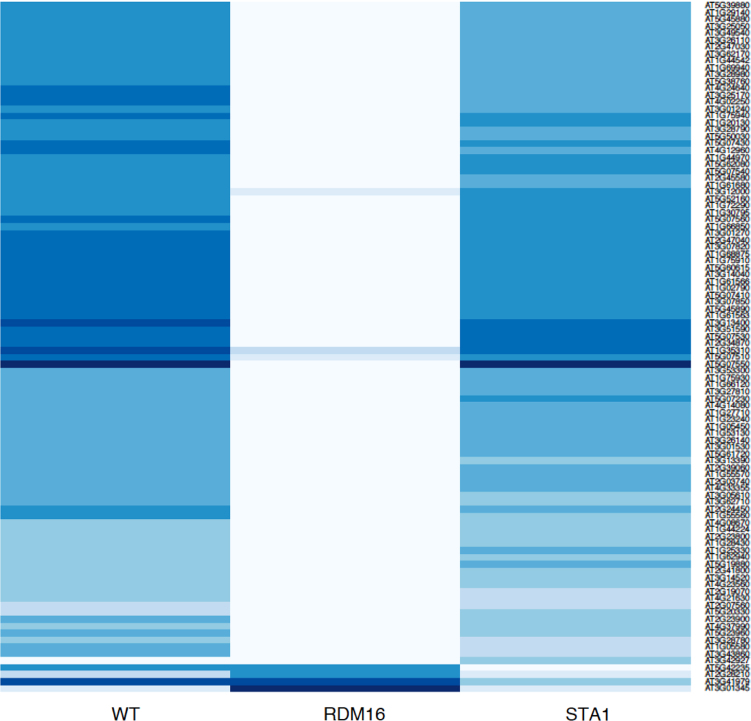
Depicts expression profile of top 100 genes that has at least one exon or intron differentially expressed in RDM16 vs STA1. The color key is given with figure.

Fig. 1, Fig. 2, Fig. 3 depict expression profile of top 100 genes that has at least one exon or intron differentially expressed in WT *vs RDM16,* WT *vs STA1,* and *RDM16 vs STA1* respectively. The color key is given with Fig. 3.

**Fig. 4:**
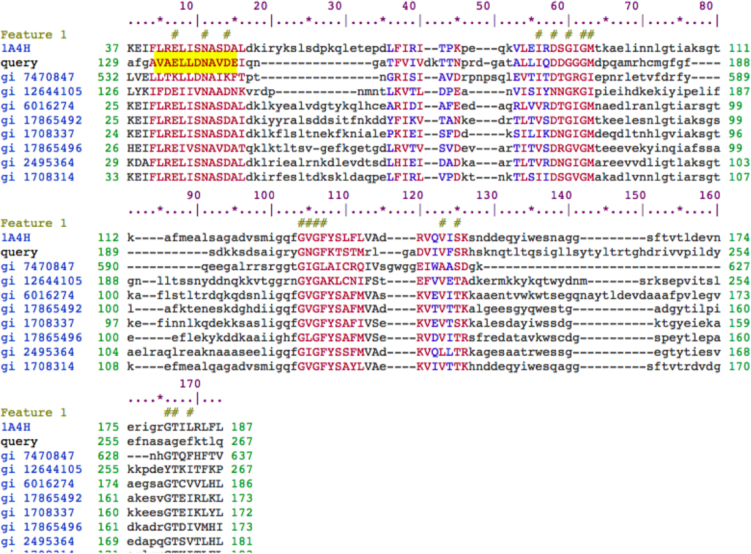
Alignment of *MORC6* protein to the ATPase-C family members that have conserved three ATP binding sites at 8, 11 and 14th position of the alignment. There are few more ATP binding sites at 55–65, 104–107, 123–125, 166–169 but may not be contributing in the ATP binding since co factor binding site is only available in the protein sequence that is coded by exon4 in *MORC6* (region highlighted in yellow).

Fig. 4: Figure shows the alignment of *MORC6* protein to the ATPase-C family members that have conserved three ATP binding sites at 8, 11 and 14th position of the alignment. There are few more ATP binding sites at 55–65, 104–107, 123–125, 166–169 but may not be contributing in the ATP binding since co-factor binding site is only available in the protein sequence that is coded by exon4 in *MORC6* (region highlighted in yellow).

**Fig. 5:**
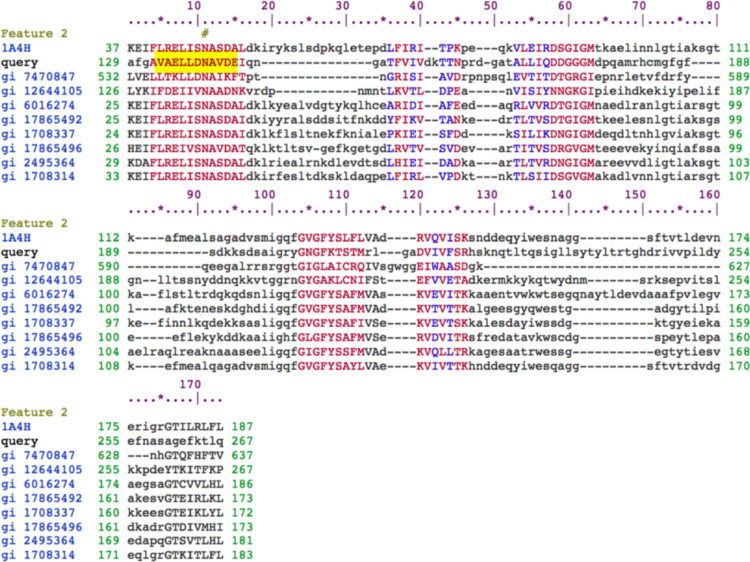
Alignment of *MORC6* protein to the ATPase-C family members that have conserved Mg^2+^ binding site at 11^th^ position of the alignment. Highlighted (yellow color) query sequence shows the protein sequence that is coded by exon4 in *MORC6*. ASP (D) and ASN (N) are essential amino acid for Mg^2+^ binding but do not contribute in it (Jorgensen et al. [7]).

Fig. 5: Figure shows the alignment of *MORC6* protein to the ATPase-C family members that have conserved Mg2+ binding site at 11th position of the alignment. Highlighted (yellow color) query sequence shows the protein sequence that is coded by exon4 in *MORC6*. ASP (D) and ASN (N) are essential amino acid for Mg^2+^ binding but do not contribute in it [7].

## 2 Experimental design, materials and methods

The experiment contains RNA-Seq samples in three conditions; WT (wild type), mutant RDM16 and STA1. The raw data were downloaded from Gene Expression Omnibus (GEO) with accession number GSE44635. The alignment of the reads were done using TopHat2 pipeline [2] (Table 1) and the reads were counted *via* featurecount function in Rsubread package [4]. We used edgeR in order to find the differentially expressed exons and introns [6]. Fig. 1, Fig. 2, Fig. 3 were prepared using in-built functions in R. The alignment of the *MORC6* protein to ATPase-C family members was done using ClustalX software [3]



Color key used in expression profiles of genes in different contrasts (for fig. 1, 2 and 3).

## Figures and Tables

**Table 1:** Summary of the TopHat2 alignment. (Values are in millions).

**Sample**	**Pairs**	**Aligned pairs (%)**	**Multiple alignments (%)**	**Discordant alignments (%)**	**Concordant pairs (%)**
*WT*	25.74	24.10 (93.6%)	1.51 (6.2%)	0.02 (0.1%)	24.08 (93.5%)
*RDM-16*	26.62	24.93 (93.7%)	1.69 (6.7%)	0.02 (0.1%)	24.91 (93.6%)
*STA1*	26.56	24.90 (93.8%)	2.15 (8.5%)	0.02 (0.1%)	24.88 (93.7%)
