# Supplementary material for: Data on RDM16 and STA1 regulate differential usage of exon/intron in RNA directed DNA Methylation pathway
Source: Data Brief. 2017 Apr 8;12:261–8. doi: 10.1016/j.dib.2017.03.050 (PMC5403763; doi:10.1016/j.dib.2017.03.050)
Supplement: Supplementary file 1 — Supplementary material [file mmc1.docx]

**Conflict of Interest**

We wish to confirm that there are no known conflicts of interest associated with this publication and there has been no significant financial support for this work that could have influenced its outcome.

We confirm that the manuscript has been read and approved by all named authors and that there are no other persons who satisfied the criteria for authorship but are not listed. We further confirm that the order of authors listed in the manuscript has been approved by all of us. On behalf of all authors, I hereby submit the revise manuscript for your consideration in the journal

Dr. Ravi Datta Sharma,

Assistant Professor,

Amity Institute of Biotechnology (AIB),

Amity Institute of Integretive Science and Health (AIISH),

Amity University Haryana, Panchgaon, NH-8, Gurgaon, India 122413
